# A minireview on nanoparticle-based sensors for detection of coronaviruses

**DOI:** 10.4155/bio-2021-0006

**Published:** 2021-08-31

**Authors:** Elaheh Rahimpour, Farzaneh Lotfipour, Abolghasem Jouyban

**Affiliations:** ^1^Pharmaceutical Analysis Research Center & Faculty of Pharmacy, Tabriz University of Medical Sciences, Tabriz, 5165665811, Iran; ^2^Food & Drug Safety Research Center, Tabriz University of Medical Sciences, Tabriz, 5165665811, Iran; ^3^Biotecnology Research Center, Tabriz University of Medical Sciences, Tabriz, 5165665811, Iran; ^4^Faculty of Pharmacy, Near East University, PO box 99138, Nicosia, North Cyprus, Mersin 10, Turkey

**Keywords:** coronavirus, nanoparticle, sensor

## Abstract

Coronaviruses (CoVs) are a class of viruses that cause respiratory tract infections in birds and mammals. Severe acute respiratory syndrome and Middle East respiratory syndrome are pathogenic human viruses. The ongoing coronavirus causing a pandemic of COVID-19 is a recently identified virus from this group. The first step in the control of spreading the disease is to detect and quarantine infected subjects. Consequently, the introduction of rapid and reliable detection methods for CoVs is crucial. To date, several methods were reported for the detection of coronaviruses. Nanoparticles play an important role in detection tools, thanks to their high surface-to-volume ratio and exclusive optical property enables the development of susceptible analytical nanoparticle-based sensors. The studies performed on using nanoparticles-based (mainly gold) sensors to detect CoVs in two categories of optical and electrochemical were reviewed here. Details of each reported sensor and its relevant analytical parameters are carefully provided and explained.

Coronaviruses (CoVs) cause infection in mammals’ and birds’ upper respiratory and gastrointestinal systems. It leads to illnesses ranging from the common cold to more severe diseases [1]. CoV is a ssRNA virus whose name comes from the Latin word corona, meaning crown or halo [2]. Its protein is short with 76–109 amino acids with an average genome size of almost 30 kb [3]. There are mainly four genera CoVs such as alpha, beta, gamma and delta which each of them is included several viruses. All of them have a zoonotic origin. The alpha and beta groups are derived from bats. The gamma and delta groups originate from avian and pig gene pools. More details about these groups of viruses are reviewed by Kumar *et al.* [4]. CoVs are likely to emerge in humans due to periodic spillover events and common cross-species infections. Once in humans, the virus has the likelihood to spread from human to human and finally caused a pandemic disease. The Middle East respiratory syndrome (MERS), Severe acute respiratory syndrome (SARS) and SARS-CoV-2 are known as pathogenic human viruses that broke out in the last decades. Simultaneously with the outbreak of these viruses, researchers had made great efforts to find accurate and real-time techniques for their early diagnosis. Various sensors (based on commonly used methods such as virus culture, ELISA, polymerase chain reaction (PCR), western blots and serological antibody detection methods) and nanosensors have been reported for CoVs detection so far. Reviewing all researches is essential and can become a database for finding more sensitive, reliable methods. Jalandra *et al.* [5] reviewed the developed sensor and biosensors to detect and diagnose SARS-CoV-2. They classified various sensors into seven classes, namely:

-  PCR-based detection: a molecular biology method employed to study gene expression at the transcript level.
-  Antibody-based detection: an analytical method that identifies the formation of an antigen-antibody complex and converts this to a conclusive read-out.
-  Aptamer-based detection: a method based on using aptamers, small-sized single-stranded artificial nucleotides (RNA or DNA) with 10–100 nucleotides, which bind to various target analytes with high specificity and affinity.
-  CRISPR-based approach: a biotechnological technique for genome editing.
-  Molecularly imprinted polymer-based detection: the molecular imprinting method is based on the selectively binding of the host components to the target molecules.
-  Microarray-based detection: a microarray consisted of carefully selected viral sequences coupled to a random amplification step, which provides a highly broad-reaching and unbiased diagnostics method.
- Loop-mediated isothermal amplification (LAMP)-based diagnostics method: the rapid amplification of DNA with high simplicity and specificity at a fixed temperature.

PCR and antibody-based diagnostics are dominated methods in SARS-CoV-2 detection due to easy to use and less time taking procedures. But alternative technologies such as LAMP, RT-LAMP, CRISPR, etc. are under development and may hit the diagnostic market in the future. Generally, all sensors comprise recognition elements and transducers applied as detection machinery of PCR or ELISA-based methods. The main principle is trapping the target and converting responses to signals [6]. Many categories of sensors have been discussed in reports based on energy source, structure and materials. In other classification based on materials used for diagonalization, there are bulk and nanostructure materials. As nanotechnology significantly improves most technologies and industries such as information technology, transportation, medicine, energy, environmental and science food safety [7], the inclusion of nanomaterials in sensing platforms of viruses also improves and optimizes their sensing capability, sensitivity and selectivity. Nanosensors can be defined as sensing devices with at least one of their sensing dimensions up to 100 nm. Although, mostly nanomaterials with spherical shape are used in the nanosensors reported for immunoassays, some materials in other forms can also be applied in the production of nanosensors, including nanoscale wires (due to their high ability for detection sensitivity), carbon nanotubes (due to their very high surface area), thin films, metal and metal oxide nanoparticles (owing to their superior physico-chemical, spectral and optical characteristics) and polymer nanomaterials [8]. The progress in nanosensors can be achieved over the improved performance of current nanosensors or designing newer nanosensors based on novel mechanisms [9]. Thanks to their unique properties, nanoparticles become ideal materials in the sensing field particularly, in disease diagnostic by electrochemical and optic instruments. Furthermore, nanotechnology has shown improved uses in biosensing by minimizing sensor elements into sizes that increase the S/N ratio. This process is mainly significant for procedures that are planned to happen at the device’s interface [10].

The current work aims to review developed methods based on nanoparticles for the detection of CoVs. Due to the importance of these types of viruses in this period, detection of other viruses by nanoparticles-based methods is excluded and the literature search was restricted to these viruses. The current emerging COVID-19, as a newly discovered coronavirus with an increasing death, is a significant global concern. As all CoVs belong to the family Coronaviridae and they have a similarity in their structures, it is hoped that this review can be a practical reference for the researchers to find a reliable and real-time technique for COVID-19 detection and diagnostic. However, it should be said that recently, some strong reviews have been published by Drobysh *et al.* [11] about affinity sensors for the diagnosis of COVID-19 and by Dronina *et al.* [12] about designing DNA sensors. In the following of these works, the present work cover the sensors based on the nanomaterials for detecting CoVs in two categories of optical and electrochemical methods. The studies reviewed here were obtained by searching the SCOPUS database with the keywords of: ‘coronavirus,’ ‘infectious bronchitis virus (IBV),’ ‘MERS,’ ‘SARS,’ ‘porcine epidemic diarrhea,’ ‘detection’ and ‘nanoparticles’. As a result, 30 articles precisely related to the subjects were found for the given keywords. Figure 1 shows the number of papers published in the last years with this subject. Briefly, explanations for each report are provided in the following sections.

**Figure 1.**
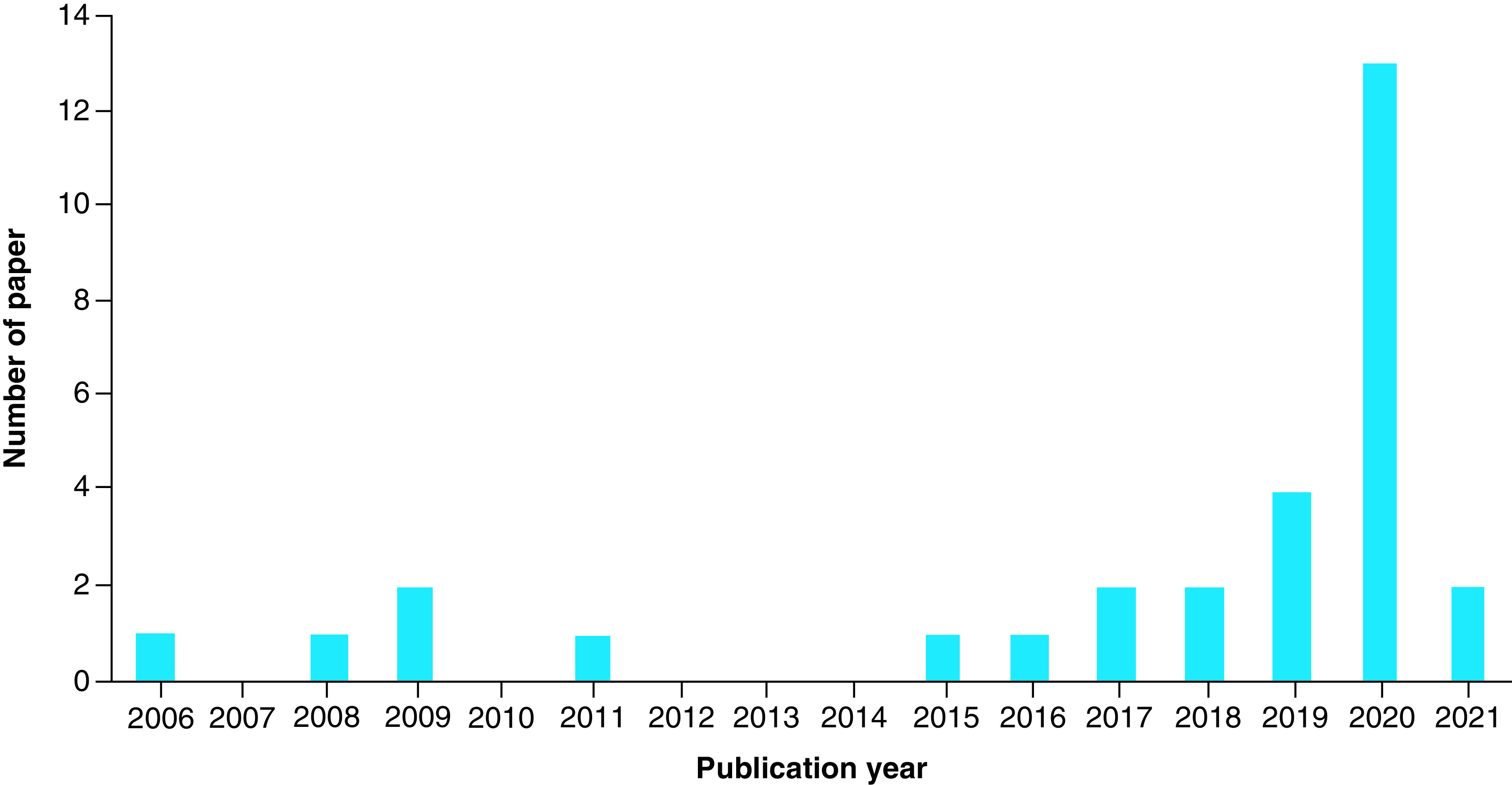
The bar chart of the number of papers by the year of publication.

## Developed nanoparticle-based methods for detection of coronavirus

The reported nanoparticle-based detection methods for CoVs are classified under the two main classes of optical and electrochemical strategies. The reported methods are summarized in Table 1, and further details for each method are given in the following sections.

**Table 1.** Characteristics of included studies on nanoparticles based sensors for determination of coronavirus.

Coronavirus type	Sensor	Sample	Linear range	Detection limit	Ref.
MERS	Aggregation based method by Au NPs conjugated with thiolated ssDNA probe	–	–	1 pmol.μl^-1^	[13]
MERS	Aggregation based method by citrate capped Ag NPs	–	20–1000 nmol.l^-1^	1.53 nmol.l^-1^	[14]
SARS-CoV-2	Agglomeration based method by ASO capped AuNPs	Oropharyngeal swab	0.2–3 ng/μl	0.18 ng/μl	[15]
IgM antibody against the SARS-CoV-2 virus	Lateral flow assay strips based on AuNP	Serum	–	–	[16]
Anti-SARS-CoV-2 IgM and IgG	Selenium nanoparticle-based LFIA	Plasma	–	–	[17]
IgG antibody against SARS-CoV-2 virus	Au NPs based LFIA	Serum	>0.8 mg/ml	–	[18]
PEDV	ICA with Au NPs-mAb	–	–	5.9 × 10^3^ TCID_50_.ml^-1^	[19]
IBV	ICS with Au NPs-conjugated antibodies	–	–	10^4.4^ EID_50_	[20]
IBV	Chiroimmunosensor composed of CAu NPs and carboxyl-capped CdTe QDs	Chicken blood	10^2^–10^4^ EID.50 ml^-1^	47.91 EID.50 ml^-1^	[21]
IBV	Immunoreaction in the presence of antibody-conjugated Zr QDs and MP NPs	–	79.15–10,000 EID.50 μl^-1^	–	[22]
SARS	Fluorescence probe composed of DyLightTM 649 labeled with secondary antibodies conjugated with Au NPs	Serum	0.1–1000 pg.ml^-1^	–	[23]
Anti-SARS-CoV-2 IgG	Fluorescence LFIA probe composed of Eu nanoparticles labeled with mouse anti-human IgG antibody	Serum	–	–	[24]
SARS-CoV-2	mRT-LAMP-nanoparticles based - LFB	Oropharynx swab	–	12 copies (for each detection target) per reaction	[25]
COVID-19	Au/FBG probe decorated with GO	Salvia	–	1.6 × 10^3^ copies/ml	[26]
SARS	Reflective biosensor based on Au NPs	–	–	–	[27]
SARS	Immunoreaction in the presence fusion proteins capped Au NPs	–	–	–	[28]
PEDV	Nano PCR assay with Au NPs	–	–	2.7 × 10^-6^ ng.μl^-1^	[29]
PEDV	Nano PCR assay with Au NPs	–	–	Feld strain: 6.10 × 10^4^ copies.μl^-1^ Attenuated strains: 7.30 × 10^5^ copies.μl^-1^	[30]
SARS	Nano PCR assay with silica coated superparamagnetic nanoparticles	–	–	2.0 × 10^3^ copies	[31]
SARS-CoV-2	RT-PCR combined with Au NPs aggregation optical-based method	Nasopharyngeal, nasal or throat swabs			[32]
SARS-CoV-2	Plasmonic photothermal biosensors by Au nanoislands	–	–	0.22 pmol.l^-1^	[33]
MERS HCoV	Electrochemical immunosensor by carbon electrode modified with Au NPs	Nasal samples	0.001–100 ng.ml^-1^ 0.01–10,000 ng.ml^-1^	–	[34]
PEDV	Electrochemical immunosensor by GCE modified with Au NPs/molybdenum disulfide/reduced graphene oxide nanocomposites	–	82.5–1.65 × 10^4^ TCID_50_ ml^-1^	–	[35]
IBV H120 strain	Voltammetry with Au NPs-assisted signal amplification	–	1.56 × 10^-9^ 1.56 × 10^-6^ μmol.l^-1^	2.96 × 10^-10^ μmol.l^-1^	[36]
SARS	Electrochemical immunosensor by carbon electrode modified with Au NPs	–	2.5–50 pmol.l^-1^	2.5 pmol.l^-1^	[37]
SARS-CoV-2	Voltammetry based on p-sulfocalix[8]arene (SCX8) functionalized graphene (SCX8-RGO), Au and Fe_3_O_4_ nanoparticles	Sputum, throat swabs, urine, plasma, feces, oral swabs, whole blood and saliva	10–17 to 10–12 mol.l^-1^	3 amol.l^-1^	[38]
COVID-19	The chemiresistors modified with functionalized Au NPs	Exhaled breath	–	–	[39]

Ag NP: Silver nanoparticle; Au NP: Gold nanoparticle; ASO: Antisense oligonucleotide; FBG: Fiber Bragg grating; GCE: Glassy carbon electrode; GO: Graphen oxide; IBV: Infectious bronchitis virus; ICA: Immunochromatographic assay; ICS: Immunochromatographic strip, mAB: Monoclonal antibody; MERS: Middle East respiratory syndrome; mRT-LAMP: Multiplex reverse transcription loop-mediated isothermal amplification; SARS: Severe acute respiratory syndrome; LFB: Lateral flow biosensor; LFIA: Lateral flow immunoassay; PCR: Polymerase chain reaction; PEDV: Porcine epidemic diarrhea virus; QD: Quantum dots; RT-PCR: Reverse transcription-PCR; TCID_50_: Median tissue culture infectious dose.

## Optical method

Optic nanoparticle based-sensors determine the reflective index change of the transducer by the formation of a complex between recognition element and target. These nanoparticle-based sensors can be categorized into two groups of direct optic nanoparticle-based sensors; signal generation is via a complex formation on the transducer surface. In contrast, the indirect optic nanoparticle based-sensors are mainly designed with different labels to diagnose the binding and extend the signal. Optic nanoparticle-based sensors may identify the presence of viruses by the following mechanisms: nanoparticles can be conjugated with a ssDNA (a specific strand that can be coupled with desired viruses) and provides a nanoprobe. In the absence of target DNA/RNA, the presence of an electrolyte induces nanoparticle aggregation, which can be detected by color change (changing in absorbance wavelength). Whereas, in the presence of target DNA/RNA, hybridization of ssDNAs on the surface of the nanoparticles with target DNA/RNA causes dsDNA – nanoparticles complex and prevents them from salt-induced aggregation. So, no color changing is observed in the presence of target DNA/RNA; (2) fluorescent nanocrystals with immunoreaction (antibody-antigen reaction) mechanism can also be used to detect viruses. Herein, the antibodies are placed on solid supporting materials such as microplate wells or beads and following a competitive or sandwich immunoreaction, labels are connected to desired analytes or surface of the transducer; determination of analytes is performed by measuring the fluorescent label’s specific activity. In this method, numerous kinds of nanomaterials, including quantum dots, upconversion nanomaterials and nanoclusters as fluorophores, come to work, enabling more stable, sensitive. Accurate detections by solving small Stokes shifts, poor photostability, fast photobleaching, aggregation and high binding rates to plasma protein for fluorescent nanocrystals mechanism; a reflective biosensor that changes in the optical reflectance properties such as the size of nanoparticles are used as a signal for virus detection. The reflective index variation in these sensors is related to the binding or the reaction of biomolecules and bioreceptors at the nanoparticle surface; plasmonic nanoparticles on a microchip is functionalized with target antibody that in the presence of antigen and antibody-antigen immunoreaction, surface plasmon resonance (SPR) properties of nanoparticles varied. Variations in SPR of plasmonic nanoparticles after target binding can be detected by SPR spectroscopy.

Based on mechanism (1), Kim *et al.* [13] reported a label-free spectrophotometry method for MERS coronavirus determination using gold nanoparticles (Au NPs). They used citrate capped Au NPs conjugated with thiolated ssDNA probes that can be aggregated in the positive electrolyte (0.1 mol.l^-1^ MgCl_2_) and absence of analyte. The hybridization of ssDNAs on the NPs surface with target DNA results in the formation of dsDNA -Au NPs and prevents Au NPs from salt-induced aggregation using disulfide induced self-assembly complex. This procedure caused a significant color change and a red-shift in the localized surface plasmon resonance (LSPR) spectrum proportionally MERS coronavirus concentration with a limit of detection (LOD) of 1 pmol.μl^-1^. Similar work was performed by Teengam *et al.* [14] for the determination of MERS coronavirus oligonucleotide. In their study, a paper-based method was used for DNA or RNA diagnosis based on nanoparticle aggregation using pyrrolidinyl peptide nucleic acid (acpcPNA). The acpcPNA probe has a positive charge due to lysine at C-terminus and leading to citrate capped silver nanoparticles aggregation in the absence of complementary DNA or RNA. In the presence of target DNA, the production of the anionic DNA-acpcPNA complex causes disaggregation of the silver nanoparticles due to the electrostatic repulsion and leads to a visible color change. This method can be used to determine MERS oligonucleotide in the range of 20–1000 nmol.l^-1^ with a LOD of 1.53 nmol.l^-1^.

**Figure 2.**
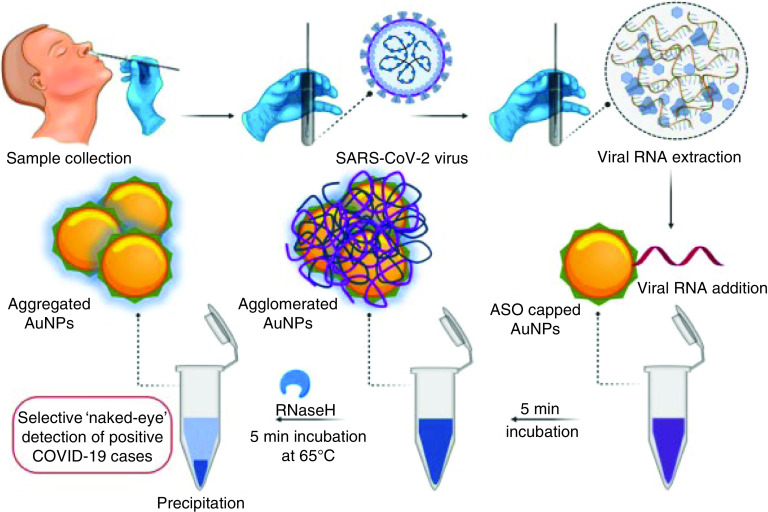
Antisense oligonucleotides capped gold nanoparticles for the selective ‘naked-eye’ diagnose of SARS-CoV-2 RNA. Further permissions related to the material excerpted should be directed to the American Chemical Society. Reproduced with permission from [15]. © American Chemical Society (2020).

Moitra *et al.* [15] reported a colorimetric method based on Au NPs for detecting positive COVID-19 cases during 10 min from the isolated RNA samples. Nanoparticles are capped with thiol modified antisense oligonucleotides (ASOs) specific for N-gene (nucleocapsid phosphoprotein) of SARS-CoV-2. The modified Au NPs agglomerate in the presence of its target RNA sequence of SARS-CoV-2 and thus show a change in its SPR in following amplified with adding RNaseH and the visually detectable precipitation of Au NPs (Figure 2). This method can be used to determine SARS-CoV-2 RNA in the concentration range of 0.2–3 ng/μl with a LOD of 0.18 ng/μl. A lateral flow immunoassay (LFIA) method for IgM antibodies against the SARS-CoV-2 virus was developed by Huang *et al.* [16], utilizing Au NPs for preparing Au NP- lateral flow assay strips, the SARS-CoV-2 nucleoprotein was immobilized on a membrane for sample trapping and antihuman IgM was conjugated with Au NPs to produce a detecting reporter. The strips performance was investigated on COVID-19 patient serum samples. The developed method obtains results within 15 min and needed only 10–20 μl serum for each experiment. Other nanoparticles LFIA kits are selenium nanoparticle-based LFIA for rapid diagonalization of anti-SARS-CoV-2 IgM and IgG in plasma samples [17] and Au NPs based LFIA for diagonalization of IgG antibody against SARS-CoV-2 virus in serum samples [18].

**Figure 3.**
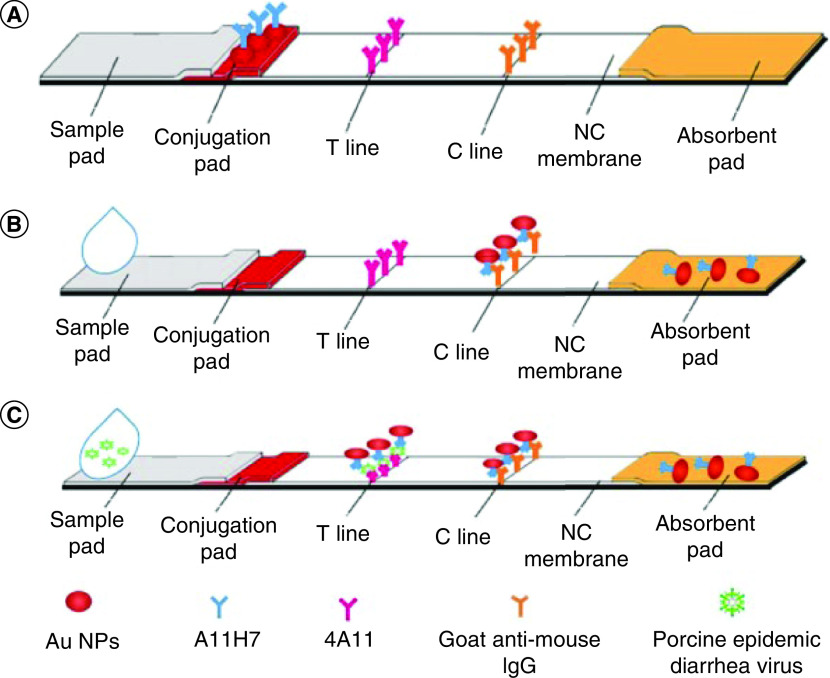
SA developed real-time probe for diagnosis of PEDV. **(A)** Schematic representation of the obtained ICA. **(B)** Negative reaction. In negative samples, no red line on the T-line was formed. **(C)** Positive reaction in positive spiked samples, a red line was observed on the T-line. ICA: Immunochromatographic assay; T-line: Test line. Reproduced with permission from [19]. © The Authors (2019).

Bian *et al.* [19] reported a new immunochromatographic assay (ICA) for real-time identification of the porcine epidemic diarrhea virus (PEDV). ICA was a sandwich-type probe (Figure 3) which is comprised of nitrocellulose membrane, polyvinyl chloride plate, sample pad, conjugate pad and absorbent pad. Phosphate buffer solution, goat antimouse IgG and diluted monoclonal antibodies (mAb) were dispensed on specific areas of nitrocellulose membrane as the control line and test line (T-line) and Au NPs-mAb was loaded onto the conjugate pad. The strip can be used after a suitable pretreatment. Cline always shows a red line due to specific antigen-antibody binding pairs. The red line at the T-line was only evident in the positive PEDV sample tracking by image J. LOD of the technique was reported to be 5.9 × 10^3^ TCID_50_/ml. A comparison between the developed ICA and reverse transcription-PCR (RT-PCR), BIONOTE test strip was performed to confirm the applicability of the developed ICA for real-time diagonalization of PEDV. Similar work was performed by Liu *et al.* [20] for the determination of avian IBV. As the previous reports, Au NPs-conjugated antibodies attach to the desired antigen. The complexes are then bound to the support matrix by unlabeled antibodies attached to the matrix. IBV was detected based on virus-specific mAbs with a LOD of 10^4.4^ EID_50_.

**Figure 4.**
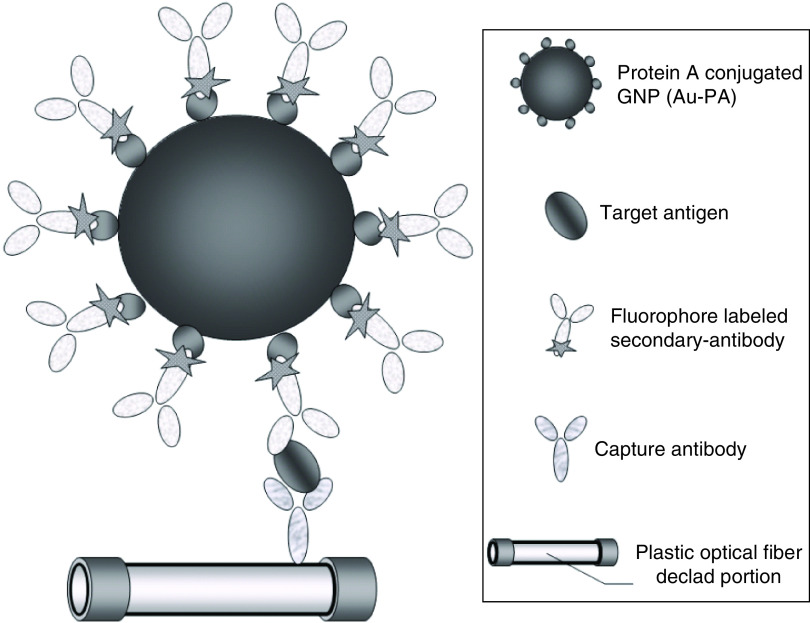
A representation of GST detection mechanism by the sandwich complex and fluorescence probe. Reproduced with permission from [23]. © Elsevier (2009).

Based on mechanism (2), Ahmed *et al.* [21] reported a chiroimmunosensor composed of star-shaped chiral gold nanohybrids (CAu NPs) and carboxyl-capped CdTe quantum dots (QDs) for the determination of IBV in chicken blood samples. Chiral-based optical sensing experiments were performed by adding antigen conjugated CAu NPs and antigen conjugated QDs into circular dichroism cuvette containing various concentrations of IBV. Self-assembly techniques of CAu NPs create asymmetric plasmonic chiral nanostructures, result in the extension of the spectral range of the circular dichroism response in the coupling with the QD excited state. The chiroptical response of IBV identification linear in the range of 10^2^–10^4^ EID/50 ml with a LOD of 47.91 EID/50 ml. In another study [22], this research group introduced aqueous soluble chiral zirconium QDs (Zr QDs). It used them for the optical detection of IBV. They employed a hetero structuring magnetoplasmonic (MP) and fluorescent nanocrystals using immunoreaction for diagnosing. After the preparation of ZrQDs and MP NPs, they were conjugated with an IBV antibody. At this point, there is no attraction between them. By adding the analyte, antibody-conjugated Zr QDs and MP NPs will form an assembled nanohybrid structure that an external magnet can extract. The photoluminescence intensity of nanohybrids can be recorded and used for the determination of IBV concentration. The analytical response obtained by this method was linear in the IBV concentration range of 79.15–10,000 EID/50 μl. Huang *et al.* [23] proposed a combination of a sandwich immunoassay with the LSPR technique for the determination of SARS nucleocapsid protein (glutathione S-transferase [GST]) in the serum samples. A polymethyl methacrylate was used as an optical fiber in this study. The polymethyl methacrylate fiber was cleaned, and the capture antibody (anti-N-1 mAb) was immobilized on its surface. The fluorescent probe also consists of DyLightTM 649 labeled with secondary antibodies (anti-N-2 mAb) conjugated with Au NPs. The fluorophore with maximum wavelength at 674 nm was excited by the enhanced localized electromagnetic field close to the Au NPs. Its luminescence intensity changes in the presence of GST were used for its determination in the range of 0.1–1000 pg.ml^-1^. The schematic illustration of GST determination by the LSPR coupled fluorescence method is shown in Figure 4.

Chen *et al.* [24] developed a lanthanide-doped nanoparticles-based LFIA for diagnosing anti-SARS-CoV-2 IgG in serum samples. They synthesized Eu^3+^ nanoparticles by miniemulsion polymerization, functionalized them by mouse antihuman IgG antibody and rabbit IgG and fixed them on an LFIA strip. A 100-μl of diluted serum samples (1:1000) was added on a strip and moved from the sample pad to the absorbent pad strip; during the migration, anti-SARS-CoV-2 IgG bind with the desired antibody. Its fluorescence was monitored using the portable fluorescence reader with λ_ex_ and λ_em_ of 365 and 615 nm, respectively. However, it should be noted that this method cannot be improved from semiquantitative to reliable quantification owing to the lack of the official anti-SARS-CoV-2 IgG standard. Zhu *et al.* [25] reported a lateral flow biosensor (LFB) based on nanoparticles combined with a multiplex reverse transcription loop-mediated isothermal amplification (mRT-LAMP) for SARS-CoV-2 detection. The N genes and ORF1ab of SARS-CoV-2 are amplified in a reaction and detected by the nanoparticle-based LFB. Fluorescein was coupled with the ORF1ab primer set. Digoxigenin was coupled with the N primer set. The mRT-LAMP system formed various fluorescein -/ digoxigenin – and biotin-attached duplex amplicons that LFB can detect through biotin/streptavidin interactions. The limit of detection for the COVID-19 mRT-LAMP-LFB approach was reported to be 12 copies per reaction.

Based on mechanism (3), Samavati *et al.* [26] used an Au/FBG probe decorated with graphene oxide (GO) for COVID-19 virus diagnosis in patients’ saliva. Deviations of the light wavelength and its intensity (in comparison with healthy saliva sample) after adding the probe to the saliva sample confirm the infection by the virus. The spike protein of CoV bind to the GO surface at 1-Ethyl-3-(3-dimethylaminopropyl)carbodiimide (EDC)/N-hydroxysuccinimide (NHS) activating agent forms an extra layer over the GO. It changes the surrounding refractive index of the sensor, which can be detected by an optical method. This method can be used for patients with the COVID-19 virus in the early infection phase with 1.6 × 10^3^ copies/ml. Park *et al.* [27] developed a reflective Au NPs biosensor for determination SARS. For the preparation of a colloidal crystal-coated chip, monodisperse silica particles were coated on a glass substrate and then functionalized with 3-aminopropyltriethoxy silane that can interact with various biomolecules and metal (Au NPs in this study) by the formation of a covalent bond. Photonic colloidal crystals can be used for optical detection of protein–metal binding by a shift in wavelength of optical spectra. The response to changes in the refractive index is resulting from the variation in the thickness and diameter of nanoparticles on the chip surface in the presence of the analyte. Optical images of colloidal crystal patterns were obtained using a CCD camera mounted on a microscope. This research group in the other study [28] and implement the mechanism (4) prepared a poly (dimethylsiloxane) microfluidic channels on the Au NPs surface and were employed for antigen-antibody studies. Fusion proteins could be easily attached on the Au NPs surface. The microfluidic channels were analyzed by SPR imaging. The surface plasmon band of nanoparticles moved to a larger wavelength as the anti-SARS coronavirus envelope protein (SCVme) antibodies were attached to the fusion proteins on the Au NPs. Moreover, the anti-SCVme antibodies attaching to its antigen changed the solution color to purple, that could be easily visualized with naked eyes. This method can be used for the efficient diagnosis of SARS coronavirus. Recently, Plikusiene *et al.* [40] published a comprehensive article and they used a total internal reflection ellipsometry method for detection of SARS-CoV-2. Although in this article, they used a Au bulk disc for determination and its methodology is behind of the present study; but they have an interesting discussion on kinetics and thermodynamics of interaction between immobilized SARS-CoV-2 nucleoprotein and specific antibodies immobilized on Au disc. Its measurement principal can be a good reference for previously mentioned studies that is conduced by plasmonic nanoparticles for determination of coronaviruses.

Some other reports for the determination of CoVs which are classified as optic methods are PCR-gel electrophoresis-UV methods. In these procedures, nanoparticle-assisted PCR (nanoPCR) as a new class of PCR modified with nanoparticles is used for making copies of a DNA sample; gel electrophoresis is used for separation and UV light was employed as a detector. Yuan *et al.* [29] used a nanoPCR approach with Au NPs for the determination of PEDV. The lower detection limit of this method was 2.7 × 10^-6^ ng.μl^-1^ for PEDV RNA and no cross-reaction was reported with other viruses. They showed that nano-PCR assay is 100-fold more sensitive than a conventional RT-PCR method. Zhu *et al.* [30] used a similar method for distinguishing field strains and attenuated strains of PEDV. The LOD of the NPs-assisted RT-PCR for the field strain was 6.10 × 10^4^ copies.μl^-1^, and that of the conventional RT-PCR method was 6.10 × 10^6^ copies.μl^-1^. For the attenuated strains, the detection limit of the NPs-assisted RT-PCR was 7.30 × 10^5^ copies.μl^-1^ and that of the usual RT-PCR method was 7.30 × 10^6^ copies.μl^-1^. Gong *et al.* [31] were also used a combination of modified silica coated superparamagnetic nanoparticles and PCR-based methods for the determination of SARS coronavirus gene with a LOD of 2.0 × 10^3^ copies. Karami *et al.* [32] used a combination of RT-PCR and Au NPs aggregation optical-based method for determination of SARS-CoV-2 in nasopharyngeal, nasal or throat swabs sample. For determination, viral RNA is isolated from the samples and reverse transcribed to cDNA. Finally, in the presence of the palindromic linker constructed as a SARS-CoV-2 RNA sensor, the specific regions of cDNA amplified using PCR. In the presence of the desired target, the linker sensor is cleavaged by the DNA polymerase in the amplification procedure. Finally, an aliquot of the PCR products is mixed with the single-component solution and a single-component assembly of Au NP-core spherical nucleic acids. After 5–10 min incubation at room temperature, the colorimetric response due to nanoparticles aggregation can be readily observed by the naked eye.

Qiu *et al.* [33] used a plasmonic photothermal sensor for the diagonalization of COVID-19. Photothermal based sensor are the sensors in which the signal results in nonradiative relaxation (e.g., heat) of absorbed energy during absorption of optical radiation in samples. Plasmonic nanoparticles with LSPR properties lead to an enhancement in the local electromagnetic field and consequently, result in strong light absorption or scattering. So, plasmonic nanoparticles serve as ‘light-activated nanoscopic heaters’ for photothermal usages [34]. The reported photothermal sensor by Qiu *et al.* [33] is a combination of the plasmonic photothermal (PPT) effect and LSPR sensing transduction. 2D Au nanoislands on an Au nanofilm chip are modified with cDNA receptors for sensitive diagonalization of the desired sequences from SARS-CoV-2 through nucleic acid hybridization. The thermoplasmonic heat is generated on Au nanoislands chip after illumination at frequency of plasmonic resonance of the nanoparticles. The localized PPT heat is able to increase the temperature of *in situ* hybridization. A high-power 532 nm laser diode was employed for PPT heating by illuminating the Au nanoislands chips in the normal incident angle. In addition to LSPR sensing, a white light sensing beam produced using an light emitting diodes (LED) source was selected. After polarization, BK7 prism was used to couple the incident radiation to the Au nanoislands–dielectric interface at an inclined nominal incident angle of 72° and excited the local electromagnetic fields in the next to the Au nanoislands by the Kretschmann configuration. The plasmonic resonance wavelength for LSPR sensing transduction was 580 nm. The local plasmonic responses were retrieved from the attenuated total reflection spectral interferograms recorded by a spectrometer which can be varied by hybridization with various concentrations of selected sequences from SARS-CoV-2. The developed method exhibits high sensitivity with LOD of down to 0.22 pmol.l^-1^ toward the desired SARS-CoV-2 sequences and allows reliable diagonalization of the specific target in a multigene mixture.

## Electrochemical method

The main principle in the electrochemical sensors is the reaction with the chemical solutions and showing an electrical signal proportional to the concentration of analyte. The electrochemical method uses amperometry, potentiometry, cyclic voltammetry and impedimetry for the determination of an analyte. Electrochemical biosensors for detecting the viruses are categorized in four classes including antibody-based, aptamer-based, antigen-based and nucleic acid-based sensors. The electrochemical techniques are good choice for point of care viral detection owing to their high selectivity and sensitivity, simple operation, portability, low cost and rapid determination procedure.

While optical detection approaches have traditionally dominated novel strategies, electrochemical devices have a main benefit including the development of reagent-free detection uses [41]. Antibody-based or antigen-based sensors have some advantages like specific and high sensor. However, denaturation and the high cost are their main disadvantages. Aptamer-based sensors have exceptional advantages such as cost-effective and reproducible production methods, long-term shelf storage at room temperature and a high flexible structure [42]. For nucleic acid-based sensors, high sensitivity and ability to recognize wide range of targets are the main benefits. However, they are influenced by environmental conditions (like temperature and pH).

**Figure 5.**
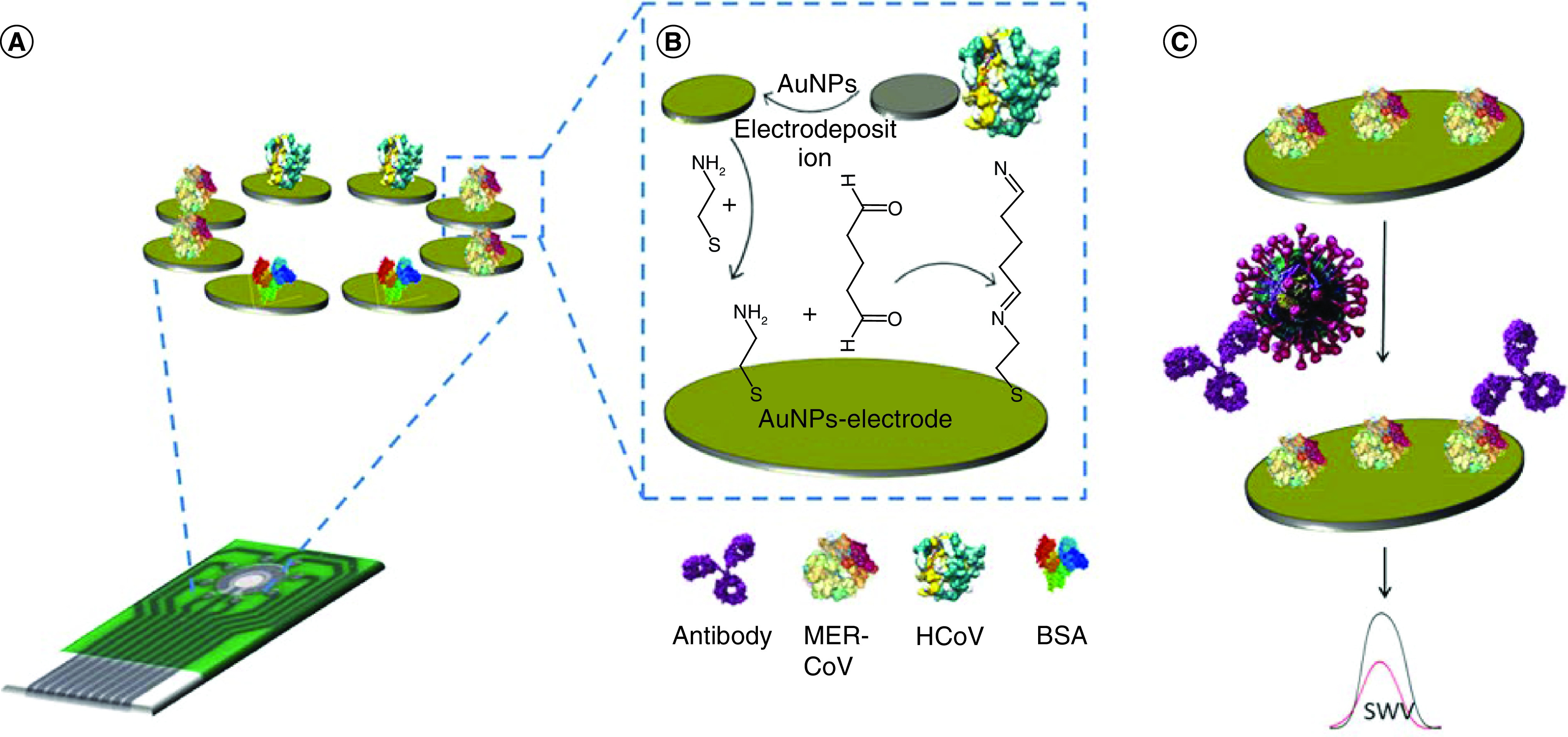
A schematic representation of designing of the MERS coronavirus immunosensor and the diagnostic procedure. **(A)** Immunosensor array chip. **(B)** The immunosensor preparation procedure. **(C)** Virus detection procedure of the competitive immunosenso. Reproduced with permission from [35]. Springer-Verlag GmbH Austria, part of Springer Nature (2019).

For antibody based sensors, Layqah and Eissa [35] reported a competitive electrochemical immunosensor for the diagonalization of MERS coronavirus in nasal samples. The biosensor is based on the indirect competition between the immobilized MERS coronavirus protein and free virus in the sample for a constant concentration of antibody which is added to the sample. The identification was achieved by recording the reduction peak current of ferro/ferricyanide redox couple after each experimental run. The antibody attaching to the fixed protein caused to a decrease in the square wave voltammetry reduction peak current. This reduction in the peak current is ascribed to the electrode surface coating with the bulky sized antibodies with a molecular weight of approximately 150 kDa. This surface coverage with the antibody retards the ferro/ferri cyanide redox couple reaching the conductive surface and subsequently decreases efficiency of the electron transfer and a decrease in the current. They used an Au NPs modified carbon electrode with immobilized recombinant spike protein S1 on its surface as a biosensor. Figure 5 demonstrates the designing of the MERS coronavirus immunosensor and the diagnostic procedure. They reported that the electrode array can be used for multiplexed identification of different CoVs. This method can be used for the determination of MERS and HCoV in the range of 0.001–100 ng.ml^-1^ and 0.01–10,000 ng.ml^-1^, respectively. Li *et al.* [43] developed another electrochemical immunosensor for the detection of PEDV. They used a GCE modified with Au NPs/molybdenum disulfide/reduced graphene oxide nanocomposites as a working electrode. Then mAB of PEDV-2C11 was also bound to the electrode’s surface. The determination of PEDV concentration was performed by quantifying the variations in the charge transfer resistance of the electrode before and after the immunoreaction between antigen-antibody by hexacyanoferrate(II)/(III) as the redox probe. This method can be determined PEDV concentration within the range of 82.5–1.65 × 10^4^ TCID_50_ ml^-1^.

**Figure 6.**
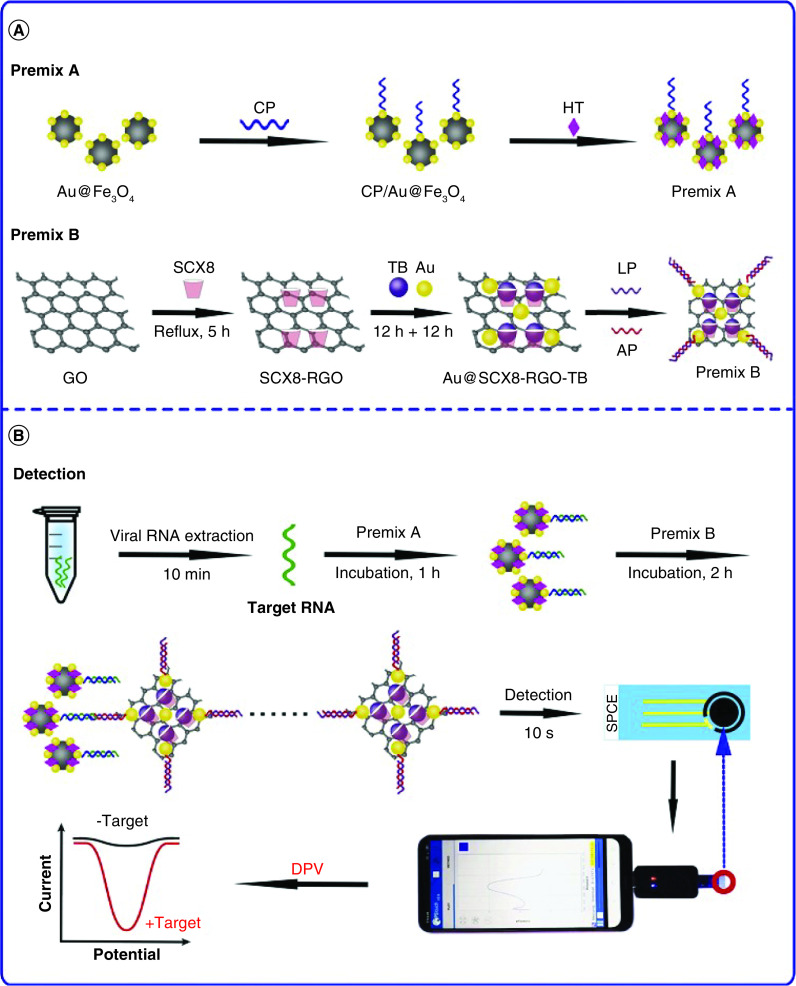
An illustration of SARS-CoV-2 determination utilizing the electrochemical biosensor. **(A)** Prepare of premix A and B. **(B)** Steps of electrochemical determination by employing a smartphone. Reproduced with permission from [38] © Elsevier (2021).

For nucleic acid based sensors, Yang *et al.* [36] reported an Au NPs-assisted signal amplification method for quantification of IBV H120 strain. Herein and based on the differences of S1 protein in various IBV strains, a hybridized dsDNA-RNA was constructed. S1 nuclease hydrolyzed the residual ssDNA, the amount of DNA becomes equal to the amount of virus RNA. Subsequently, the target DNA is isolated from DNA-RNA hybridized double strand using heating. The target DNA may slightly hybridize using probe 2 modified on the electrode surface and probe 1 on the surface of Au NPs. Au NPs are placed on the surface of the electrode and the abundant DNA on Au NPs’ surface adsorb the molecules of hexaammineruthenium (III) chloride in the solution to generate a significant analytical response. The voltammetric method shows a detection range of 1.56 × 10^-9^ to 1.56 × 10^-6^ μmol.l^-1^ with the detection limit of 2.96 × 10^-10^ μmol.l^-1^ for IBV H120 strain. Martinez-Paredes *et al.* [37] reported a hybridization-based nanosensor for the determination of the SARS virus. Au NPs are formed *in-situ* using a constant current intensity on the carbon electrode and an immobilization and transduction surface role. The formation of the sensing phase was performed with 3′-thiolated oligonucleotide probe, and then a blocking step with casein was performed. The hybridization was done at room temperature by pouring 3′-biotinylated oligonucleotide target solutions on the surface of the genosensor for 1 h and then washing the probe and performing another reaction with streptavidin labeled to alkaline phosphatase by dropping aliquots of its solutions on the genosensor and incubation for 60 min. The washed electrode can be used for the enzymatic reaction. The detection step was performed in 3-indoxyl phosphate and silver ions solution. The 3-indoxyl phosphate, in the presence of immobilized alkaline phosphatase, generated a compound with reduced ability of silver ions into a metallic deposit that is placed where the enzymatic label alkaline phosphatase is bind. The deposited silver is electrochemically stripped into the solution and determined by anodic stripping voltammetry. The height of the stripping peak of silver depends on the concentration of alkaline phosphatase. The sensor’s response is linear in the range of 2.5–0 pmol.l^-1^ biotinylated target concentration with a LOD of 2.5 pmol.l^-1^. Zhao *et al.* [38] reported a super sandwich-type electrochemical p-sulfocalix[8]arene (SCX8) functionalized graphene (SCX8-RGO) biosensor to enrich toluidine blue for SARS-CoV-2 RNA detection in sputum, throat, swabs, urine, plasma, feces, oral swabs, whole blood and saliva. The procedures for sensor preparation are as follow: the capture probe (CP) labeled with thiol were immobilized on the surfaces of the Au@Fe_3_O_4_ nanoparticles and formed CP/Au@Fe_3_O_4_ nanocomposites; the host-guest complexes (SCX8-TB) were immobilized on RGO to form Au@SCX8-TB-RGO- label probe bioconjugate; the sandwich structure of ‘CPtarget-LP’ produced; and auxiliary probe was introduced to form long concatamers. For finding details, the schematic representation is given in Figure 6. They synthesized the artificial target of ssDNA according to the sequences of SARS-CoV-2 RNA and explored the feasibility of the biosensor. A high electrochemical signal peak was observed after incubation with the artificial target (10^-12^ mol.l^-1^). However, the differential pulse voltammetry signal was extremely weak in the absence of a target. The optimized condition for the synthesized artificial target is summarized in Figure 6. Briefly, 10 μl of samples and 50 μl of premix A were placed for 1 h at room temperature, then incubated for 2 h with 50 μl of premix B at room temperature. Subsequently, using the smartphone, the electrochemical response of toluidine blue was quantified in less than 10 s. This method can be used to detect target ssDNA in the range of 10^-17^–10^-12^ mol.l^-1^ with a LOD of 3 amol.l^-1^.

Shan *et al.* [39] reported a probe array based on nanoparticles with numerous capabilities for COVID-19 detection in exhaled breath. These probes were consisted of various Au NPs linked to some organic ligands, which produce a sensing platform with the capability of swelling or shrinking upon exposure to the volatile organic compounds (VOCs). In these platforms, the electrical conductivity is related to Au NPs. The organic film element produces the adsorption sites specific for VOCs. VOCs diffuse into the sensing platform during exposure and react with the organic part of the functional groups on the nanoparticles. These reactions lead to swelling/shrinkage in the nanomaterial film and consequently in a volume change. So, the contacts among the inorganic nanomaterial blocks change (higher/lower) related to an increase/decrease of the conductivity.

## Conclusion & future perspective

Nowadays, nanosensors have great potential in many fields, particularly in the detection of diseases. High specificity and sensitivity are the main advantages for these types of sensors that no need to spend a long time in the pre-enrichment step. Nanoparticles in a couple with different biomolecules generated probes consisting of a nanoparticle core (at various sizes and shapes) for signal readout and a surface layer of one or multiple types of biomolecules for target detection and interaction. This created a new way of using nanoparticles to compensate for the limitations of commonly used methods. In the current study, a summary of developed nanosensors for the detection and determination of CoVs is discussed based on applied methodology and instrumentation. This classification shows that most of the used methods for diagnosing this type of virus are optical methods demonstrating that the methods with simple instrumentation have higher acceptance.

On the other hand, according to Table 1, it is deduced that most nanosensors used for the detection of CoVs are Au NPs based sensors. One question that arises here is whether we cannot use other plasmonic nanoparticles instead. This can be a challenge for future works. However, significant advances in the nanosensors area indicate a promising future. The current situation with COVID-19 as a global concern shows more than ever the importance of reliable and fast viruses detection in reducing mortality. As evident from the recent COVID-19 pandemic, a rapid, low cost, non-invasive, simple and sensitive detection test is required to detect the virus early to prevent its transmission, especially in its latent phase and nanosensors show such a capability. Although limited diagnostic works were performed for CoVs detection, according to the previous data obtained from other types of viruses, nano-based sensors have proven to evoke a more sensitive response to the relevant viruses. Therefore, further research is recommended to address the nanoparticle-based sensors to study the COVID-19 diagnostic method. Colorimetric sensing, immunosensing, photoluminescence, electrochemiluminescence, electrochemical and chiroimmunosensing sensors are potential techniques to detect CoVs in the future with the aid of nanoparticles. Furthermore, the development of DNA sensor designing based on clustered regularly interspaced short palindromic repeats – Cas9 mechanism has progressed over the last years and it has been shown that these sensor for virus diagnose enhances the sensitivity of detection. This method as a universal analytical method is awarded the 2020 Nobel Prize in Chemistry. However, further and complementary works are required to accomplish the standard requirements to be approved by health authorities for use in clinical applications.

**Executive summary**

**Background**

- Coronavirus is an enveloped positive-sense ssRNA virus that causes respiratory tract infections in birds and mammals.
- The development of rapid and reliable detection methods for coronaviruses is crucial.
- Nanoparticles play an essential role in this by their high surface-to-volume ratio, and exclusive optical property enables the development of highly sensitive analytical NP-based sensors.

**Results & discussion**

- Various nanoparticle-based (mainly gold) optical and electrochemical methods have been developed to detect and quantify coronaviruses.
- Optic nanoparticle based-sensors determine the reflective index change of the transducer by the formation of a complex between recognition element and target.
- The main principle in the electrochemical nanosensors reacts with the chemical solutions and shows an electrical signal.

**Conclusion**

- Nanoparticle-based nanosensors create a new way in clinical applications to compensate for the limitations of commonly used methods.
- As evident from the recent COVID-19 pandemic, finding a rapid, lowcost, noninvasive, sensitive and straightforward detection test is required for early detection of the virus to prevent its transmission.
- Our aim in this study, reviewing almost all reported nanoparticle-based methods for the detection of this class of viruses.
